# Psychometric characteristics of DSM-5 eating disorder diagnostic criteria: support for a transdiagnostic approach

**DOI:** 10.1186/s40337-025-01512-7

**Published:** 2026-01-16

**Authors:** Evangeline Giannopoulos, Mark Hilsenroth

**Affiliations:** Derner School of Psychology, Adelphi University, Hy Weinberg Center for Psychological Services, 158 Cambridge Avenue, Garden City, NY 11530 USA

**Keywords:** Eating disorders, Adult, DSM-5, Psychometrics, Transdiagnostic

## Abstract

This study is the first to comprehensively examine psychometric characteristics of the three main DSM-5 eating disorder (ED) diagnoses (Anorexia Nervosa (AN), Bulimia Nervosa (BN), Binge Eating Disorder (BED)) amongst adult ED patients. Data was collected via an online survey from 126 licensed therapists treating an adult with an ED. Therapists provided an ED DSM-5 diagnosis for a randomly selected patient, as well as endorsed the presence of symptoms from a list of DSM criteria. Criteria endorsement varied across both the entire sample and within diagnoses. Convergence and cohesion also varied within diagnoses. The first factor analysis for DSM-5 ED criteria yielded a five-factor solution for ED criteria accounted for 78.3% of variance: (1) Binge eating, (2) Compensatory and purging behaviors, (3) Shape/weight overvaluation, (4) Drive for thinness, and (5) Absence of binging and purging. Finally, diagnostic efficiency statistics were consistent with this factor model. Overall, our findings support a new, transdiagnostic model with fear of weight gain and weight being main aspects of self-evaluation as common underlying factors amongst all EDs.

**Supplementary Information:**

The online version contains supplementary material available at 10.1186/s40337-025-01512-7.

## Abstract 2

The diagnosis of eating disorders (EDs) is currently separated by distinct categories. However, our analysis argues for more commonality across these different categories. For example, fear of weight gain and weight influencing one’s self-evaluation appears to be common across *all* categories. Certain diagnoses, including anorexia and bulimia, may benefit from more flexible descriptions, including a less strict weight requirement for the former and a less strict frequency of bingeing/purging requirement for the latter. In all, there are similarities amongst different ED categories currently viewed as distinct from one another, especially between anorexia and bulimia.

**Supplementary Information:**

The online version contains supplementary material available at 10.1186/s40337-025-01512-7.

## Introduction

The prevalence of eating disorders (EDs) has been increasing, with a 4.3% increase between the 2000–2006 and 2013–2017 time periods [1]. There has also been an increase in EDs and their severity since the COVID-19 pandemic [2]. Concurrently, there have been significant changes to ED criteria in the fifth edition of the Diagnostic Statistical Manual of Mental Disorders (DSM-5) [3]. These changes include the addition of binge eating disorder (BED), the removal of amenorrhea (lost menstrual cycle) as a criteria for anorexia nervosa (AN), and a decreased frequency of bingeing and purging required for a bulimia nervosa (BN) diagnosis, ultimately resulting in clinical diagnoses for individuals who previously did not meet formal criteria. Despite these statistics and changes, research on psychometric properties of DSM ED criteria, including specifically DSM-5 criteria, is scant.

In fact, to our knowledge there is only one DSM-5 field study of adult patients that includes an eating disorder diagnosis in its analyses [4]. This field study found a fair intraclass kappa (0.56; [5]) between two independent clinicians diagnosing BED in adult patients. Another DSM-5 field study assessed the validity and clinical utility of severity specifiers Bulimia Nervosa (BN) severity specifiers (i.e., mild, moderate, severe, or extreme, based on frequency of compensatory behaviors), and found these specifiers demonstrated predictive validity on various pre- and post-treatment measures related to BN behaviors and correlates [6]. Lydecker, Ivezaj, and Grillo [7] assessed the validity and clinical utility of Binge Eating Disorder (BED) severity specifiers. They found the specifiers did have strong validity or utility in predicting treatment response, but there were main correlational effects across different severity categories.

Prior to the DSM-5, there were some psychometric studies conducted on DSM-IV ED criteria. Williamson et al. conducted two factor analyses of DSM-IV ED criteria on a randomly divided sample of 341 ED and non-ED participants (i.e., ~ 170 participants per analysis) (2002). Their analysis resulted in three factors: (1) Binge eating, (2) Fear of fatness/compensatory behaviors, and (3) Drive for thinness. These three factors accounted for 66% of the variance in ED symptoms in their mixed sample. However, to date there has been no factor analysis of the DSM-5 ED criteria.

Several factor analyses have also been conducted on the modified ED examination questionnaire (EDE-Q), a measure of ED behaviors that overlaps significantly with individual DSM-5 ED criteria [8]. For example, a recent factor analysis of the EDE-Q (*N* = 1,981 non-clinical college students) resulted in three factors that accounted for 63.1% of variance: (1) Dietary Restraint, (2) Preoccupation and Eating Concern, and (3) Shape/Weight Overvaluation [9]. A 2012 factor analysis of the EDE-Q (*N* = 688 BED patients) resulted in similar three factors that accounted for 80.3% of variance: (1) Dietary Restraint, (2) Shape/Weight Overvaluation, and (3) Body Dissatisfaction (Grilo et al.). In addition to these factors being similar to one another, they are also similar to factors from Williamson et al. DSM-IV factor analysis [10]: The DSM-IV Factor 2 (fear of fatness/compensatory behaviors) is comparable to EDE-Q factors related to shape/weight overvaluation and the DSM-IV Factor 3 (drive for thinness) is comparable to EDE-Q factors related to dietary restraint. Further, the variance demonstrated in [10] is comparable to that demonstrated in [9], and the variance demonstrated in [11] was likely higher than these two given it was an exclusively clinical BED sample.

Factor analyses have also been conducted on versions of the Eating Disorder Diagnostic Scale (EDDS), another measure of ED symptoms that overlaps with DSM-5 ED criteria [12]. One study analyzed the factor structure of the EDDS on a sample of individuals with bipolar disorder (*N* = 1,031; [13]). Their analysis resulted in three factors: (1) shape/weight concerns, (2) binge eating behaviors, (3) compensatory behavior. The majority of studies performed factor analyses on non-English versions of the EDDS. For example, an analysis of the Korean version of the EDDS (*N* = 72 patients diagnosed with EDs) resulted in an ultimate four-factor structure: (1) body dissatisfaction, (2) binge behavior, (3) binge frequency, and (4) compensatory behavior [14]. An analysis of the Portuguese version of the measure (*N* = 185 non-clinical college students) resulted in a three-factor model, identical to that of Crow, et al. [13]: (1) Body and Weight Concerns, (2) Binge Eating Behavior, and (3) Compensatory Behaviors [15].

There have also been several latent class analyses performed on DSM ED criteria. Carr and Grilo [16] studied a sample of BED patients as per DSM-5, and found a two-class model: BED and BED with cognitive eating disorder and depressive symptoms. These classes had significant differences from one another in body image concerns (i.e., overevaluation of weight/shape and dissatisfaction with weight/shape items on EDE), distress about binge eating, and depression symptoms. Though the difference between these two classes in frequency of binge episodes was also significant, it was a smaller effect than the other differences listed. Castellini et al. [17] studied a sample of clinical and subthreshold ED patients as per the DSM-IV, and found a five-class model: severe binging, moderate binging, restricted eating, binge and moderate purging, and binge and severe purging. They also found significant differences between these classes on several psychopathology and treatment measures, such as the severe binging as well as binge and severe purging classes having significantly higher eating concern, shape concern, and weight concern scores than the other classes. At a six-year follow up with the same sample, they instead found a three-profile solution of binge eating, binge eating and purging, and restricted eating. Finally, Crow et al. [18] examined a sample of ED patients as per DSM-IV at an outpatient clinic, finding a six-class solution each characterized by moderate levels of binge eating and purging,highest levels of binge eating and vomiting; moderate levels of binge eating and purging and highest levels of laxative, diuretic, and enema use; individuals of “overweight” or “obese” BMI who endorsed binge eating; individuals with a BMI less than 19 who were highly likely to endorse excessive fear of weight gain; and individuals below a BMI of 19 with an uncommon endorsement of fear of weight gain.

White and Grilo performed diagnostic efficiency analyses on the five proposed indicators of a binge eating episode, prior to the official classification of BED in the DSM-5 [19] in a mixed sample of 916 ED patients (BED and BN) and non-clinical controls. They found all five indicators to have good predictive value. More specifically, eating alone because embarrassed had the highest positive predictive power (PPP = 0.80), meaning there was an 80% probability a participant had “binge eating” when this indicator was present. The strongest overall correct classification predictive and kappa values were for eating when not hungry and eating alone because embarrassed. The former saw a total predictive value (TPV) of 0.82, meaning that there was an 82% chance this criteria accurately predicted a “binge eating” diagnosis, and a kappa of 0.55, meaning this criteria alone accurately predicted binge eating 55% of time, even when corrected for chance. The latter saw a TPV of 0.82 and kappa of 0.48. Again, like with factor analyses, we are not aware of any diagnostic efficiency analyses for criteria of BED since the publication of DSM-5, and none for the DSM-5 criteria for AN and BN.

The present study is unique and distinctive in that it is the first to comprehensively examine psychometric characteristics of AN, BN, and BED DSM-5 criteria sets amongst adult ED patients, as per therapist report of their clinical practice with these patients. It is also the first to conduct a factor analysis on DSM-5 ED diagnoses. The objectives are: (1) Establish basic psychometric characteristics (frequency, convergence, internal consistency) for DSM-5 ED criteria, (2) Determine a factor structure amongst DSM-5 ED diagnostic criteria, and (3) Determine diagnostic efficiency (sensitivity, specificity, positive predictive power, negative predictive power, overall correct classification rate, kappa) of DSM-5 ED diagnostic criteria. Given prior ED psychometric research, we hypothesized that diagnostic kappa values would be fair (0.4–0.59) to good (> 0.60) [5], as well as that BED would have a high level of diagnostic efficiency. Given prior factor analysis research on DSM-IV and the EDE-Q, we hypothesized that our DSM-5 ED criteria factor structure would contain several of the five previously observed factors, in no particular order: (1) Binge eating, (2) Shape/weight overvaluation (psychological experience of weight loss), (3) Drive for thinness (physical process of weight loss), (4) Compensatory and purging behaviors, and (5) Body dissatisfaction. Additional analyses were largely exploratory and thus do not have specific hypotheses.

## Method

### Participants

Participants were 126 mental health clinicians currently treating an adult with an ED. Detailed characteristics of clinicians are provided in Table 1; Note that the same sample was used in Giannopoulos & Hilsenroth [20]. This sample comprises clinicians from across North America with various disciplines and theoretical orientations, which we believe increases the ecological validity of our results.

**Table 1:** Demographic and clinical information of therapist sample (N = 126)

Variable	n	%	M	SD
Gender				
Female	116	92.1%		
Male	10	7.9%		
Other	0			
Mean age (years)			42.31	10.68
Discipline				
Psychiatrist	1	.8%		
Psychologist	51	40.5%		
Social Worker	25	19.8%		
Master’s-level therapist	48	38.1%		
Other	1	.8%		
Clinicians’ years in practice			14.89	10.68
Theoretical orientation				
Cognitive-behavioral	63	50%		
Psychodynamic	26	20.6%		
Interpersonal	14	11.1%		
Family-systems	9	7.1%		
Humanistic-existential	6	4.8%		
Other	8	6.3%		

### Procedure

Approval for carrying out the following procedure was first obtained from Adelphi University’s Institutional Review Board. Participants were recruited from several professional electronic forums accessed through the Academy for Eating Disorders, the International Association of Eating Disorders Professionals, the National Eating Disorder Association, Division 29 (Psychotherapy) of the American Psychological Association, Society for Psychotherapy Research, Adelphi University Derner School of Psychology, PsychologyToday.com, and LinkedIn’s Eating Disorder Clinicians and Researchers. Participants were recruited primarily from the United States and Canada, but also internationally to allow for a geographically diverse sample. Participants received an invitation via their professional e-mail or LinkedIn account. The invitation included the link to a Qualtrics survey, as well as a solicitation flier that described the study and what clinicians would be asked of. Clicking on the survey link provided immediate access on participants’ own technological devices. The survey began with informed consent, which assured clinicians that they could end their participation at any time and encouraged them to contact the principal investigator with any questions. By clicking *I Consent*, clinicians proceeded to anonymously fill out the survey (no IP addresses collected). In addition to anonymity, clinicians were informed that all data would be stored electronically on a secure server and a password-protected website accessible to only the principal investigators. No compensation was provided for participants. To comply with informed consent, our survey allowed for leaving answers blank without penalty; nonetheless, data were complete for our sample. A validity check was also included during the survey, and only participants who correctly answered this validity item (“Please answer ‘always’ for this question”) were then included in analyses. The data were collected over a six-month period, beginning in March 2022. To increase response rates, we posted three waves of solicitations, including the initial one in March, another two months following in May, and another three months following in August. These efforts resulted in a sufficient response rate of 126 therapists. With this procedure, this study met all ethical and safety-monitoring standards.

To complete the survey, clinicians were asked to select an adult patient 18 years or older who was diagnosed with an ED currently in their care for a minimum of eight sessions. Therapists were instructed to select only one patient in light of therapist effects, wherein therapeutic process and ultimate outcome is better with certain therapists than others, regardless of patient characteristics and treatment needs (e.g., [21]). If clinicians were to report on more than one patient, their unique therapist effects could skew the overall sample. Therapists were also asked to select this one patient at random by consulting their calendar and selecting the last patient they saw that week that met criteria, to minimize selection biases, and with the prerequisite of having worked together for a minimum of eight sessions, to ensure they had good clinical knowledge of the patient (e.g., Giannopoulos & Hilsenroth [20]; Thompson-Brenner & Westen [22]).

Data and materials from this study will be made available upon request of the first author.

### Measures

#### Demographic information

Participants were asked six demographic questions pertaining to discipline, gender, age, years of practicing as a clinician, years licensed, and primary theoretical orientation. Participants also provided patient age and gender.

#### Categorical ED diagnosis

Clinicians were asked to report their patient’s initial DSM-5 eating disorder diagnosis at the start of treatment. Clinicians chose one of the following DSM-5 categorical ED diagnoses: AN, BN, BED, Other Specified Eating Disorder (write-in text box option), and Unspecified Eating Disorder (write-in text box option).

#### ED criteria symptom checklist based on the DSM-5

Participants were then separately administered a randomized, exhaustive checklist of 13 symptoms as per DSM-5 criteria across AN, BN, BED, unspecified eating disorder, and other specified eating disorder diagnoses to determine the presence or absence of these symptoms at the start of treatment. All items on the checklist can be found in Supplemental Table 1. This checklist is similar in format to the Eating Disorder Assessment for DSM-5 (EDA-5), which lists individual symptoms and asks if each is present in a “yes/no” format. The EDA-5 has demonstrated reliability and validity in a virtual format [23].

### Analysis plan

We utilized G*Power version 3.1 to conduct a power analysis (see [24]). According to Cohen [25], for product moment analyses, an R^2^ of 0.10 is considered a small effect size, 0.30 medium, and 0.50 large. Given our ultimate sample of 126 clinicians and an alpha error probability of 0.05, we had 20% probability to detect a small effect size, 93.1% probability to detect a medium effect size, and 99.10% probability to detect a large effect size.

Regarding basic psychometric statistics, endorsement frequencies for each ED criteria were determined. Adjusted item-to-scale (point-biserial) correlations were then calculated for each criteria comprising the three ED diagnoses. ED criteria were analyzed as per clinician-reported diagnosis (e.g., analyze AN criteria as per clinician-reported AN diagnosis), and the individual criterion being evaluated was excluded from its total parent ED score. For example, in analyzing the criterion of fear of weight gain in relation to the AN scale, this criterion would be removed from the overall scale that usually comprises BMI < 85%, fear of weight gain, and weight being a main aspect of self-evaluation. Coefficient alphas amongst criteria that define each disorder and kappa values between clinician-rated categorical DSM-5 diagnosis and clinician-endorsed individual ED DSM-5 checklist criteria were also calculated. Intercorrelations amongst DSM-5 ED diagnoses as per clinician-endorsed criteria were calculated between each ED diagnosis.

In addition to basic psychometrics, a principal component factor analysis with varimax rotation was performed for all ED criteria. Orthogonal rotation specifically was performed to preserve the independence and simplicity of factors. A factor analysis versus latent class analysis was performed to fill the gap in the existing literature that does not contain a DSM-5 factor analysis, and therefore have a current point of comparison to other prior, related factor analyses (e.g., ED diagnostic measures like the EDE-Q that are commonly used in clinical practice for diagnostic purposes).

Finally, efficiency statistics of ED criteria were calculated. Efficiency statistics included: sensitivity (SN), specificity (SP), positive predictive power (PPP), negative predictive power (NPP), overall correct classification rate (OCC), and Kappa.

## Results

### Sample characteristics

Solicitation resulted in a sample size of 126 experienced mental health clinicians who each reported on a single ED adult patient currently in their care. Table 2 presents full patient demographic data, as reported by clinicians. Patients that were described averaged 29.74 years old (*SD* = 11.69). The majority (*n* = 117; 92.9%) were female and primarily diagnosed with AN (*n* = 55; 43.7%). Other patients were diagnosed with BED (*n* = 24; 19%), BN (*n* = 21; 16.7%), other specified ED (*n* = 20; 15.9%), and unspecified ED (*n* = 5; 4%). One clinician did not report a specific diagnosis for their patient. Clinicians reported working with their patients for on average over a year and a half (*M* = 19.10 months).

**Table 2:** Reported demographic information of patient sample (N = 126)

	n	%	M	SD
*Gender*				
Female	117	92.9		
Male	8	6.4		
Other	1	.8		
Mean age (years)			29.74	11.69
*Clinician-rated DSM Initial Dx*				
Anorexia Nervosa (AN)	55	43.7		
Bulimia Nervosa (BN)	21	16.7		
Binge ED (BED)	24	19		
Unspecified ED	5	4		
Other Specified ED	20	15.9		
Months in Tx			19.10	19.09

### Basic psychometric characteristics of DSM-5 ED criteria sets

Table 3 provides endorsement frequencies for the individual DSM-5 ED criteria in the sample. These frequencies provide base rate information regarding the assignment/occurrence of each criterion. Notably, fear of weight gain and weight being the main aspect of self-evaluation were the two highest endorsed criteria (each > 80%) across the entire sample. Compensatory episodes was the third highest endorsed criterion (> 50%) across the entire sample. For AN specifically, fear of weight gain (89%), weight being the main aspect of self-evaluation (91%), and a BMI < 85% (76%) were all highly endorsed criteria. However, despite being a required criteria for AN DSM-5 diagnosis, 24% of clinicians did not endorse the specific individual criterion BMI < 85% for the patients they had previously assigned a categorical diagnosis of AN to at the start of treatment. For BN, binge eating and compensatory episodes (each 91%) were the most endorsed criteria, followed by fear of weight gain and weight being the main aspect of self-evaluation (each 81%). Again, despite being a required criterion for BN DSM-5 diagnosis, 19% of clinicians did not endorse the specific individual criterion weight being the main aspect of self-evaluation for the patients they had previously assigned a categorical diagnosis of BN to. Marked distress about binge eating (91%), binge characterization (86%), and purging episodes (86%) also had notable endorsements for BN. Finally, for BED, binge eating episodes (91%), binge characterization (91%), and marked distress about binge eating (83%) were the most endorsed criteria. Despite being a required criteria for BED DSM-5 diagnosis, 17% of clinicians did not endorse the specific individual criterion marked distress about binge eating for the patients they had previously assigned a categorical diagnosis of BED to. As with both other EDs, BED also saw high endorsement for fear of weight gain (71%) and weight being the main aspect of self-evaluation (79%).

**Table 3:** Endorsement frequencies (%) for the DSM-5 ED individual criteria by clinician diagnoses

	AN	BN	BED	Total
	(*n* = 55)	(*n* = 21)	(*n* = 24)	(*n* = 126)
Criteria #				
1—BMI < 85%	76	19	8	41
2—Fear of weight gain	89	81	71	84
3—Weight self-evaluation	91	81	79	87
4—Binge eating episodes	11	91	92	42
5—Compensatory episodes	62	91	17	56
6—< 1 compensatory/month	13	24	17	18
7—Distress binge eating	20	91	83	48
8—Binge characterization	9	86	92	40
9—10% weight reduction	55	29	8	38
10—Binge, low freq	7	91	75	37
11—Compensatory, low freq	53	91	17	52
12—Purging episodes	29	86	8	34
13—< 1 binge per month	11	24	21	14

Table 4 provides adjusted item-to-scale (point-biserial) correlations for DSM-5 EDs. AN and BED each had one adjusted correlation below 0.30, while all other criteria analyzed, including all criteria required for a BN diagnosis, had adjusted correlations above 0.30 [26]. More specifically, AN’s BMI criteria had an adjusted item scale correlation of 0.26, versus correlations of 0.51 and 0.58 for fear of weight gain and weight being the main aspect of self-evaluation criteria, respectively, indicating adequate convergence for these two criteria related to the psychological experience of weight for these AN patients. BED’s marked distress about binge eating criteria had an adjusted correlation of 0.27, versus correlations of 0.72 for each binge eating episode occurrence and binge characterization criteria, indicating adequate convergence amongst the objective experience of a binge. Finally, there was adequate convergence for an overall BN diagnosis. Table 5 also provides coefficient alphas for each ED. BED had an alpha coefficient above 0.70, indicating adequate cohesion amongst these overall criteria sets; Both AN and BN had a coefficient below 0.70, indicating a lack of cohesion amongst these overall criteria sets [26].

**Table 4:** Adjusted item-to-scale correlations, coefficient alphas and kappas for DSM-5 ED clinician-rated criteria and diagnoses

DSM-5 ED clinician diagnoses			
	AN (*n* = 55)	BN (*n* = 21)	BED (*n* = 24)
Criteria #^a^			
1—BMI < 85%	.26		
2—Fear of weight gain	.51		
3—Weight self-evaluation	.58	.30	
4—Binge eating episodes		.42	.72
5—Compensatory episodes		.42	
7—Distress binge eating			.27
8—Binge characterization			.72
Coefficient Alpha	.61	.56	.71

^a^Criteria 6, 10–13 not included on this table as not included in diagnoses reported on

**Table 5:** DSM-5 eating disorders (ED) individual criteria factor structure^a^ (N = 126)

	Factor loadings				
DSM-5 ED criterion	I	II	III	IV	V
1—BMI < 85%				.767	
2—Fear of weight gain			.877		
3—Weight self-evaluation			.912		
4—Binge eating episodes	.950				
5—Compensatory episodes		.846			
6—< 1 compensatory/month					.863
7—Distress binge eating	.835				
8—Binge characterization	.928				
9—10% weight reduction				.807	
10—Binge, low freq	.903				
11—Compensatory, low freq		.880			
12—Purging episodes		.744			
13—< 1 binge per month					.707
Eigenvalue	3.477	2.215	1.730	1.441	1.318
Variance	26.7	43.8	57.1	68.2	78.3

^a^Extraction Method = Principal Component Analysis, Rotation Method = Varimax with Kaiser Normalization

The principle component analysis of the 13 ED criteria revealed five factors with eigenvalues greater than 1 and suitable locations on a scree plot. Further, the data demonstrated a Kaiser–Meyer–Olkin value of 0.74 and a Bartlett’s test significance of < 0.001, both indicating appropriateness for factor analysis. Table 5 presents the five-factor solution for ED criteria accounting for 78.3% of cumulative variance. Factor one contained primary loadings for ED criteria 4 (binge eating episodes), 7 (marked distress about binge eating), 8 (binge characterization), and 10 (binge episodes, low frequency), and was interpreted as corresponding to *binge eating (26.7% of variance)*. Factor two contained primary loadings for ED criteria 5 (compensatory episodes), 11 (compensatory episodes, low frequency), and 12 (purging episodes). This factor was interpreted as a reflection of *compensatory and purging behaviors (17.1% of variance)*, which are essential to a diagnosis of BN. Factor three included primary loadings for ED criteria 2 (fear of weight gain) and 3 (weight main aspect of self-evaluation), reflecting the psychological *shape/weight overvaluation (13.3% of variance)*, an essential piece of an AN (and, in part, BN) diagnosis. Factor four included ED criteria 1 (BMI < 85%) and 9 (10% reduction in weight), reflecting the physical experience of weight loss and *drive for thinness (11.1% of variance)*, the other essential piece of an AN DSM-5 diagnosis. Finally, Factor five included ED criteria 6 (< 1 compensatory per month) and 13 (< 1 binge episode per month), reflecting the *absence of binging and purging (10.1% of variance)*. This factor could arguably, along with Factors 3 and 4, represent the restricting subtype of AN.

Table 6 presents the diagnostic efficiency statistics for the individual ED criteria. In this clinical sample of ED outpatients, criterion 1 (BMI < 85%) for an AN diagnosis had the best overall classification rate, meaning that it was the criterion that had the best rate of correctly classifying AN patients, the best Kappa, meaning that it had the best level of agreement corrected for chance with an accurate AN diagnosis, the strongest specificity, meaning it had the strongest ability to correctly identify individuals without AN, and the strongest positive predictive power, meaning it had the strongest probability to predict AN. This criterion is parallel to Factor 4 above, suggesting the utility of a criterion related to physical weight in differentially diagnosing AN. Overall, all three BED criteria (4—binge eating episodes, 8—binge characterization, and 7—marked distress about binge eating) also had strong diagnostic efficiency. All three of these criteria for BED had some of the highest sensitivity and specificity values, meaning the highest ability to predict individuals with BED and without BED, respectively; high overall classification rates, meaning high rates of correctly classifying BED patients; and high Kappa values falling just below those of criterion 1, meaning that they had high levels of agreement corrected for chance with an accurate BED diagnosis. These findings are parallel with Factor 1 above, suggesting this factor is an important part of a differential BED diagnosis. Both exclusively BN criteria (4—binge eating episodes and 5—compensatory episodes) notably had sensitivity, specificity, overall classification rates, and kappa values comparable to BED criteria. Criterion 5 specifically is parallel to Factor 2 above, indicating the role of this factor in distinguishing a BN diagnosis from other EDs, including BED, with which it shares some features. Criteria related to the psychological experience of weight (2—fear of weight gain, 3—weight main aspect of self-evaluation) notably had weak Kappa values, meaning there were weak levels of agreement between these criteria and an accurate diagnosis of AN and/or BN. This finding indicates the ubiquitous nature of these criteria across the entire ED sample. These criteria also reflect high endorsement frequencies across the whole sample and Factor 3 above, further suggesting this factor is non-specific to unique ED diagnoses and rather underlies all EDs.

**Table 6:** Diagnostic efficiency statistics for the DSM-5 ED individual criteria (N = 126)

Anorexia nervosa criteria	SN/SP	PPP/NPP	OCC	Kappa
1—BMI < 85%	.64/.86	.81/.82	.82	.63
2—Fear of weight gain	.89/.20	.46/.70	.50	.08
3—Weight self-evaluation	.91/.16	.46/.69	.48	.06
**Bulimia Nervosa Criteria**				
3—Weight self-evaluation	.81/.11	.16/.75	.23	-.03
4—Binge eating episodes	.91/.68	.36/.97	.71	.36
5—Compensatory episodes	.91/.51	.27/.96	.57	.21
**Shared AN and BN Criteria**				
3—Weight self-evaluation	.88/.14	.61/.44	.59	.02
**Binge Eating Disorder Criteria**				
4—Binge eating episodes	.92/.70	.42/.97	.74	.42
7—Distress binge eating	.83/.61	.33/.94	.65	.28
8—Binge characterization	.92/.73	.44/.97	.76	.45
**Shared BN and BED Criteria**				
4—Binge eating episodes	.91/.85	.77/.95	.87	.73

*SN*, Sensitivity (ability of criterion to correctly identify individuals with a particular ED); *SP*, Specificity (ability of criterion to correctly identify individuals without a particular ED); *PPP*, Positive predictive power (probability that an individual has a particular ED when the criterion identifies as such); *NPP*, Negative predictive power (probability that an individual does not have a particular ED when criterion identifies as such); *OCC*, Overall correct classification rate (overall “hit rate” of the proportion of particular ED patients and other ED patients in the sample correctly classified by the criteria); *Kappa*, level of agreement between the defining criteria and an accurate diagnosis of a particular ED, corrected for chance agreement

Finally, intercorrelations amongst DSM-5 ED diagnoses were all significant, but ranged from small to large in magnitude of effect. Specifically, total AN criteria endorsed by clinicians were positively related to total BN criteria endorsed (*r* = 0.31, *p* < 0.001) and negatively related to total BED criteria endorsed (*r* = -0.22, *p* = 0.013). Total BN criteria endorsed were also positively related to total BED criteria endorsed (*r* = 0.62, *p* < 0.001). Additionally, kappa values indicate a good level of agreement between clinician-reported categorical DSM-5 AN diagnosis and subsequent clinician endorsement of individual criteria required to meet that diagnosis (*k* = 0.60), whereas for BN (*k* = 0.54) and BED (*k* = 0.43), kappa values indicate only a fair level of agreement between clinician-reported categorical DSM diagnosis and subsequent clinician endorsement of individual criteria for each diagnosis.

## Discussion

Our data provides information on how experienced clinicians with expertise in treating EDs make use of current DSM-5 criteria in clinical practice, and in turn offers suggestions for how these criteria can be understood and perhaps amended. Results demonstrated that several individual DSM-5 ED symptom criteria are frequently found across the three main EDs (AN, BN, BED), regardless of diagnostic category. Specifically, two criteria related to the psychological experience of shape/weight overvaluation (i.e., fear of weight gain and weight being the main aspect of self-evaluation) were highly prevalent across the entire ED sample of various diagnoses. This finding is consistent with prior research that has demonstrated the presence of these symptoms in BED patients, despite these criteria not comprising a DSM-5 BED diagnosis [16, 17, 27–29]. This finding is also consistent with Hierarchical Dimensional Taxonomy (HiTOP) of Eating Disorders studies, which have found the broad category of body image and weight concerns (e.g., Sellbom et al. [30]) and, similarly, body dissatisfaction (e.g., Forbush et al. [31]) to be relevant across all EDs. Further, the three main DSM-5 ED diagnoses demonstrated varying psychometric properties. AN and BED each had a criteria that demonstrated inadequate item convergence amongst other criteria within their diagnostic group (BMI criteria for AN, distress about binge eating criteria for BED), whereas all BN criteria demonstrated adequate convergence of individual criteria. Overall, BED demonstrated adequate internal consistency, whereas both AN and BN demonstrated inadequate internal consistency. AN had the best overall classification reliability of the three ED diagnoses (i.e., κ = 0.60), with values of all ED diagnoses ranging from fair to good (0.43 to 0.60).

Though there has been limited research on basic psychometrics of DSM EDs, a comparison to the few existing studies is worthwhile. A BED kappa value of 0.43 in the current study was still considered to be “fair” [5], but slightly lower than that of 0.56 in Regier et al. DSM-5 field trial [4]. This is despite the fact that the current study calculated kappa between the same clinician’s reported categorical DSM-5 diagnosis and subsequent endorsement of individual DSM-5 ED criteria that match this diagnosis, whereas the latter field study analyzed kappa between two independent clinician-reported categorical diagnoses. Therefore, one might have expected higher kappa reliability in this study. Instead, it seems in applied practice, experienced clinicians with specialized skills in treating ED only had a fair to good congruence between their categorical diagnoses with the individual criteria required for a DSM-5 diagnosis.

Compared to prior research, this study uniquely assessed psychometrics of all three ED diagnoses. Regarding AN specifically, the BMI < 85% criterion only had an endorsement frequency of 76% amongst patients with a clinician-reported AN diagnosis, even though it is a requirement for this DSM-5 diagnosis. This criterion did generally remain exclusive to clinician-reported AN patients, contributing to the higher kappa value of AN compared to BN and BED. However, the two other criteria required for an AN DSM-5 diagnosis (fear of weight gain and weight being the main aspect of self-evaluation) were ubiquitous across the entire sample of differential diagnoses. Given this, it may be more valuable to conceptualize a distinct AN diagnosis with a less strict weight criterion (e.g., current criterion 9 of 10% weight reduction that is relevant to Atypical AN diagnosis).

This finding related to a less strict weight criterion and Atypical AN (AAN) is consistent with clinical and research trends demonstrating the prevalence of AAN. For example, a systematic review by Harrop et al. [32] found that, in epidemiological studies, AAN was more common than AN, indicating the importance of capturing patients with this presentation in diagnostic systems. However, in clinical care setting samples, AAN was not as prevalent, suggesting barriers to care for these patients due to diagnostic thresholds and/or difficulty clinicians in care settings have classifying these patients. In fact, clinicians’ consistency in diagnosing AAN can vary depending on percentage of weight loss [33], and a qualitative study has demonstrated that clinicians in actual clinical practice believe AAN should be removed as a distinct diagnosis and considered as AN and have concerns over AAN’s vague criterion of “significant weight loss” [34]. Other studies have indeed demonstrated that there are not significant clinical differences between AN and AAN. For example, Wong and Lowe [35] found that any differences in general symptoms, such as depressive and anxiety symptoms, between AAN and AN, restricting subtype groups did not remain significant when controlling for BMI. A meta-analysis by Johnson-Munguia [36] found patients with AAN to have significantly more symptomatology for several markers than patients with AN, including shape and weight concern, drive for thinness, body dissatisfaction, and overall ED pathology.

BN demonstrated the weakest psychometric properties of all three EDs, likely because BN criteria overlap with both AN and BED diagnoses: shape/weight overvaluation is ubiquitous across the whole sample and binge eating episodes are shared with BED. Compensatory behaviors are most specific to BN, and thus a differential focus on these specific symptoms may help make a BN diagnosis more specific and have increased reliability. Further, as with AN, expansion to include lower frequency compensatory behaviors may be valuable. Finally, regarding BED, it appears binge eating occurrence in itself is the most distinctive criterion, whereas distress about binge eating and binge characterization endorsements have notable frequency in BN patients, too. These overlaps between BN and BED explain their lower kappa values, and are consistent with how these EDs are often together referred to as “binge-spectrum” disorders.

In all, it appears that clinicians in actual practice diagnose and work with patients with EDs in a way that is not strictly consistent with DSM-5 ED criteria. Some prior research, both on EDs and other psychiatric disorders, suggest a similar trend in practice. For example, a rapid review by Bryant et al. [37] found that, across multiple healthcare settings, clinicians delay or miss ED diagnoses, especially diagnoses of BED, Other Specified Feeding or Eating Disorders (which includes AAN), and sub-threshold EDs. This review also suggested that this diagnostic pattern occurs due to different EDs having similar, overlapping features to one another. Further, its findings are consistent with those related to diagnosing AAN in clinical practice, cited above, which suggest deviation from and disagreement with formal diagnostic criteria in the DSM. Further, apart from EDs, prior research has demonstrated inconsistency between DSM criteria and clinician practice for personality disorders. Westen and Arkowitz-Westen [38] found only 39.4% of a sample of patients being treated for significant personality patterns fit formal DSM-IV criteria for personality disorders. In fact, Westen and Shedler [39] argued that clinicians tended to rely more on inferences drawn from content patients share and in-session behavior than diagnostic criteria when making diagnoses, and that a sample of clinicians with years of experience and a long length of knowing patients has a higher reliability than a “small number of experts sitting at a committee table, regardless of how knowledgeable the experts are.”

A factor analysis of individual DSM-5 ED criteria revealed a five-factor model: (1) Binge eating, (2) Compensatory and purging behaviors, (3) Shape/weight overvaluation, (4) Drive for thinness, and (5) Absence of binging and purging. This model supports our hypothesis in that four of our factors are consistent with the five factors from prior research. There are also several additional similarities between the current factor model and prior analyses in the literature. Williamson et al. three-factor model of DSM-IV ED criteria, with which our sample had a comparable size (*N* = 126 ED patients in current study, *N* = 341 mixed ED patients and non-ED controls in Williamson et al. [10], first factor of binge eating parallels with our first factor of binge eating, their second factor of fear of fatness/compensatory behaviors parallels with our third factor of shape/weight overvaluation and second factor of compensatory and purging behaviors, and their third factor of drive for thinness parallels with our fourth factor of drive for thinness. Further, there are additional parallels between the current study’s factor model and prior factor analyses of EDE-Q, which includes items that overlap with DSM ED criteria. For example, Habashy et al. [9] first factor of dietary restraint parallels our fourth factor of drive for thinness, and their third factor of shape/weight overvaluation parallels our third factor of shape/weight overvaluation. Grilo et al. [11] first factor of dietary restraint and our second factor of shape/weight overvaluation have the same parallels. Note that the samples from these studies were considerably larger than the current study’s, and the former was a non-clinical sample while the latter was an exclusively BED clinical sample. Further, the former study’s diagnostic data was provided via participant self-report, whereas the latter’s was determined by experienced, EDE-Q-trained research clinicians. There are also similarities with factor analyses of the EDDS, another measure that includes items that overlap with DSM ED criteria. Our first factor of binge eating, second factor of compensatory and purging behaviors, and third factor of shape/weight overvaluation all have parallels to factors in Crow et al. [13], Bang et al. [14], and Santos et al. [15]. Finally, our factor analysis results also have parallels with results of HiTOP studies. For example, Forbush et al. [31] found a best fit, most specific model to include factors of body dissatisfaction, which shares parallels with our factors of shape/weight overvaluation and drive for thinness; bulimia, which shares parallels with our factor of compensatory and purging behaviors; restricting, which shares parallels with our factor of drive for thinness; and excessive exercise, which shares parallel with our factors of compensatory and purging behaviors and drive for thinness. Our factor analysis results do not, however, have direct parallels to the factors of negative attitudes towards obesity, cognitive restraint, social anxiety, physiological distress, insomnia, and well-being that Forbush et al. model include.

Diagnostic efficiency calculations further revealed the strongest and weakest diagnostic criteria related to these five factors. Though the current study does not have a direct parallel comparison to Dakanalis et al. [6] study on the validity and clinical utility of BN severity specifiers, the current study did see adequate convergence amongst BN criteria, similarly to how the former study saw predictive validity of severity specifiers. Findings are also consistent with White and Grilo [19] efficiency analyses on the five proposed indicators of binge eating: As they found all five indicators to have strong predictive value, with one criteria having a negative predictive power of 0.93 and two other criteria having total predictive values of 0.82, the current study found all three official BED criteria to have strong diagnostic efficiency, with negative predictor powers ranging from 0.94 to 0.97 and overall classification rates ranging from 0.65 to 0.76. Further, the current study found the BED criteria of binge characterization, the criterion that most closely reflects White and Grilo’s five indicators, to have the strongest efficiency of all BED criteria.

In all, the results of the factor and diagnostic efficiency analyses continue to suggest a transdiagnostic model for EDs. More specifically, the current study’s finding that fear of weight gain and shape/weight overvaluation being ubiquitous across our sample of diverse diagnoses suggests these criteria may be common, transdiagnostic factors to all EDs. Though the diagnosis of BED appears to be more distinct, the diagnoses of AN and BN continue to demonstrate commonalities over the domains of weight (psychological and physical) and compensatory behaviors.

Some may argue that clinician diagnoses and report of criteria are not as reliable sources of information as a formal field trial (e.g., [4]). Our sample consisted of clinicians with an average 15 years of experience who have known the patients they reported on over an extended period of time (on average 19 months). If clinicians with this level of experience working in the context in which these diagnoses and symptoms are meant to be applied are unreliable in diagnosis and symptom reporting, who is better? Trained, outside observers have limitations, too, and are likely to be less informed in their assessments than seasoned clinicians with intimate knowledge of their patients. Further, our sample includes clinicians with varying theoretical orientations and training backgrounds that roughly reflect these characteristics in actual clinical practice.

Despite this being the first comprehensive psychometric study of DSM-5 ED criteria amongst adult ED patients and its contributions regarding a transdiagnostic model, there are notable weaknesses. First, the current results are bound to the specific sample studied, which was exclusively outpatient and heavily female (both therapists and patients). There was a preponderance of AN diagnoses in the current sample, and the specific low representation of other specified eating disorders (e.g., Atypical AN). Finally, though power analyses deemed our sample size more than adequate to detect medium and large effects, our sample may be limited in detecting small effects.

Future research should expand on these weaknesses by conducting similar studies in different, more diverse, and larger samples, as well as with other diagnostic systems that have similar features to the DSM. Future research can also benefit from including individuals with sub-clinical EDs in their samples, as well as having non-diagnostic controls as a comparison group. In addition, examining which ED criteria respond more quickly to treatment interventions and are most amenable to change over the course of treatment would be valuable. Finally, it would be valuable to consider the interactions between ED diagnoses and personality structure diagnoses to further understand commonalities and differences across EDs.

## Conclusions

Overall, this first comprehensive psychometric study of DSM-5 ED criteria amongst adult ED patients suggests that there are current limitations in these criteria to diagnose specific EDs in actual clinical practice. Specifically, criteria homogeneity across the three major distinct diagnoses (AN, BED, BN) was demonstrated, and therefore consideration of a transdiagnostic model may be more useful than current guidelines. Current criteria related to the psychological experience of weight specifically seem to be underlying this transdiagnostic ED model. Specific suggestions for differential diagnoses are provided, too. Diagnostic instructions should be modified as per these suggestions to more accurately capture the range of ED profiles seen in clinical practice (i.e., less strict weight and compensatory criteria for AN and BN, respectively, and regarding BN and BED together as “binge-spectrum” disorders). These suggestions and modifications may be relevant to other diagnostic systems, as well. Though this study was largely exploratory, future replications may warrant additional diagnostic suggestions for EDs in the next DSM edition.

## Supplementary Information

Additional file 1 (DOCX 7 KB)

## Data Availability

The datasets used and/or analyzed during the current study are available from the corresponding author on reasonable request.
