# Supplementary material for: Psychometric characteristics of DSM-5 eating disorder diagnostic criteria: support for a transdiagnostic approach
Source: J Eat Disord. 2026 Jan 16;14:24. doi: 10.1186/s40337-025-01512-7 (PMC12829015; doi:10.1186/s40337-025-01512-7)
Supplement: Supplementary file 1 — Additional file 1 (DOCX 7 KB) [file 40337_2025_1512_MOESM1_ESM.docx]

**Table S1**

*ED Criteria Symptom Checklist Based on DSM-5*

Item # Item

1 BMI less than 85% of the median expected for age and gender

2 Definite fear of weight gain more than 75% of the days for at least 3 months

3 Weight and shape were one of the main aspects of self-evaluation

4 At least 4 uncontrollable binge-eating episodes per month for at least 3 months

5 At least 4 compensatory behavior episodes per month for at least 3 months

6 Less than 1 compensatory behavior on average per month during the period

7 Marked distress about binge eating

8 Binge eating characterized by 3 or more of the following: rapid eating; eating

until uncomfortably full; eating large amounts when not physically hungry; eating

alone because of embarrassment; feeling disgusted, depressed, or guilty after overeating

9 At least a 10% reduction in weight

10 At least 2 uncontrollable binge-eating episodes per month for at least 3 months or

at least 6 episodes over a shorter period

11 At least 2 compensatory behavior episodes (i.e., self-induced vomiting, laxatives,

or diuretic use, fasting, and excessive exercise to compensate for overeating) per

month for at least 3 months or at least 6 episodes over a shorter period

12 At least 4 episodes of self-induced vomiting or diuretic/laxative use for weight

control purposes per month for at least 3 months

13 Less than 1 uncontrollable binge-eating episodes on average per month during

this period
